# Taking Advantage of the Teachable Moment: A Review of Learner-Centered Clinical Teaching Models

**DOI:** 10.5811/westjem.2017.8.35277

**Published:** 2017-12-05

**Authors:** Sneha A. Chinai, Todd Guth, Elise Lovell, Michael Epter

**Affiliations:** * University of Massachusetts School of Medicine, Department of Emergency Medicine, Worcester, Massachusetts; † University of Colorado School of Medicine, Department of Emergency Medicine, Aurora, Colorado; ‡ University of Illinois at Chicago School of Medicine, Department of Emergency Medicine, Chicago, Illinois; § Advocate Christ Medical Center, Department of Emergency Medicine, Oak Lawn, Illinois; ¶ Maricopa Medical Center, Department of Emergency Medicine, Phoenix, Arizona

## Abstract

When working in a chaotic Emergency Department (ED) with competing priorities, clinical teaching may be sacrificed for the sake of patient flow and throughput. An organized, efficient approach to clinical teaching helps focus teaching on what the learner needs at that moment, incorporates regular feedback, keeps the department on track, and prevents over-teaching. Effective clinical teaching in a busy environment is an important skill for senior residents and faculty to develop. This review will provide a critique and comparison of seven structured teaching models to better prepare readers to seize the teachable moment.

## INTRODUCTION

Teaching within the Emergency Department (ED) demands a successful balance between providing efficient medical care while incorporating meaningful educational experiences for learners. Limited time, regular interruptions, institutional lack of rewards for education, and learners at different levels contribute to the challenges of clinical teaching. As a result, the atmosphere of the ED can often create an emphasis for the learner to communicate “just the facts” rather than formulating questions and discussing uncertainties.

Clinical teachers equipped with learner-centered educational strategies can create powerful learning experiences. Effective teachers identify the individual educational needs of learners, teach to those gaps, and provide feedback.^1^ Given the steady stream of undifferentiated patients with wide ranging complaints, educators can transform EDs into rich learning environments.

The goal of this paper is to present the reader with a critique of 7 teaching models to optimize learner-centered teaching in busy clinical settings. The table includes a comparative summary. Concrete examples which illustrate the use of the technique are found in Appendix 1. The teaching models share common themes including identification of learner needs, focusing relevant teaching to those needs, and the importance of feedback. The core educational theories in medical education have been previously described.^2^

## THE ONE-MINUTE CLINICAL PRECEPTOR/MICROSKILLS

### Description

The original Five-Step Microskills model of clinical teaching is commonly known as the One-Minute Preceptor (OMP).^3^ OMP is a learner-centered technique, but the momentum of the educational encounter is driven by the preceptor. It is therefore an excellent approach for novice learners and for those unfamiliar with the technique. The first three microskills identify gaps in learning, while the last three steps provide feedback. Not all steps need to be used in every encounter, and the order of the steps is flexible.

#### 1. Get A Commitment

The initial buy-in from the learner is critical, and establishes investment in the case. The learner processes information just collected from the patient, and articulates their own diagnosis or plan. One or two clarifying questions from the preceptor may be helpful but it is important to avoid over-questioning and dominating the encounter.

#### 2. Probe For Supporting Evidence

This step evaluates a learner’s knowledge and clinical reasoning. The preceptor explores the information supporting the initial commitment, assists the learner in synthesizing data, and identifies gaps in knowledge or deduction.

#### 3. Teach General Rules

This is an opportunity to teach common take-home points and rules of thumb. Preceptors provide a brief patient care pearl, focusing on principles that are easily applied to other similar cases.

#### 4. Reinforce What Was Done Right

Providing positive feedback to the learner about points of the case where they got it right reinforces competency. Feedback should be case specific and behavior focused. Preceptors can give feedback early to reduce learner performance anxiety and keep learners engaged.

#### 5. Correct Mistakes

This step should emphasize how to correct knowledge gaps or behavior related to patient care. In some cases, an actual mistake may not have occurred, but the focus is on what could have been done better or differently. The preceptor provides specific constructive or formative feedback. It’s important to choose an appropriate setting with consideration to privacy.

#### 6. Identify Next Learning Steps

This more recently added practice-based learning and improvement step is a chance for preceptors to ask a clinical question and identify resources for future study. It can also be a time for preceptors to acknowledge their own uncertainty and solve the question together with the learner.

### Literature Review

OMP is the best studied of the common clinical teaching models^4^. In studies comparing traditional teaching models and OMP, both learners and preceptors favor OMP.^5^–^9^ In a trial randomizing internal medicine residents to a one-hour OMP training session, students reported significant improvements in residents’ teaching scores and these results mirrored the residents’ self-reported improvement.^5^ In another study, a group of 116 preceptors, those utilizing OMP were more likely to emphasize higher-order thinking.^6^ Faculty viewing videos rated OMP more effective and efficient than the traditional model.^7^

In a study evaluating the effectiveness of an OMP faculty development workshop, faculty self-reported improvement in all the microskills. Positive trends in 4 of 5 microskills were noted by learners assessing participating faculty.^8^ Another study assessing the impact of an OMP faculty development workshop demonstrated an increase in amount of feedback, and increased preceptor satisfaction.^10^

OMP has been used successfully in a variety of settings. Nursing preceptors reported persistent improvement in their teaching skills.^11^ On inpatient wards, senior residents are often in a dual teacher/learner role during inpatient rotations, and OMP allows for modeling of the faculty member.^12^

### Recommendation

OMP is an intuitive, easy to apply clinical teaching model that emphasizes higher order learning such as clinical reasoning, includes feedback in every encounter, and is supported by the medical literature. It is especially helpful for novice learners and for junior faculty development.

## SNAPPS

### Description

SNAPPS aims to encourage both diagnostic and clinical reasoning in a dynamic fashion.^13^–^15^ At the core of SNAPPS is a shift in paradigm where the preceptor no longer plays the central role. Unlike OMP which is learner-centered and preceptor-driven, SNAPPS is both learner-centered and learner-driven, emphasizing autonomous learning. This conceptual interaction finds its precedent in reflective practice.^13^ More preparatory training is necessary for SNAPPS than for OMP, and it is therefore used more with senior residents.

#### Summarize

The presentation should be a concise, relevant summary of the key historical points and exam findings, not to exceed more than half of the total learner presentation.

#### Narrow

The learner should focus on the most probable 2–3 differentials. The formulation of the differential list will be driven by the learner’s baseline knowledge. While this is similar to OMP, the SNAPPS model requires the learner to commit prior to engagement of the preceptor.

#### Analyze

During this step, the learner leads an appraisal of the differential through a review of the patient’s pertinent positives/negatives to compare diagnostic possibilities. This provides the opportunity for the learner to verbalize his/her clinical reasoning.

#### Probe

This represents the unique aspect of the model - the learner leads in affirming uncertainty, rather than the preceptor asking questions to uncover gaps in the learner’s knowledge or skills. The learner then asks the preceptor questions to close their own knowledge gap.

#### Plan

A commitment is obtained from the learner through the discussion of the management plan for the patient. The preceptor is used in this step as an information resource.

#### Select

To close the teaching encounter, a focused learner-directed activity is selected to reinforce principles discussed in the case. Examples may include reading an evidence-based article, presenting a short didactic, or listening to a podcast.

### Literature Review

Users of SNAPPS expressed diagnostic uncertainty more often than comparison groups, without adding time to presentations.^14^–^17^ This led to more teaching about clinical reasoning. Acknowledging uncertainty is linked to improved critical thinking skills.^18^ While most of the literature on SNAPPS has been generated by the original authors, one study of Japanese junior residents compared SNAPPS and OMP using a simulated patient case. SNAPPS performed better at demonstrating uncertainty and was more positively rated than OMP.^19^

### Recommendation

SNAPPS is a well-studied teaching model. Compared with OMP, utilization of SNAPPS provides learners the opportunity to be more active in their learning, including questioning of the preceptor and choosing topics for asynchronous learning. While this technique can be learned at any level, SNAPPS may be most appropriate as learners become more advanced and have identified areas for self-improvement, with these “practice gaps” serving as teaching points.

## MiPLAN

### Description

Firmly grounded in constructivism and the social learning aspects of behaviorism, MiPLAN helps learners create and utilize new knowledge, and assists learners in becoming self-directed. Showcased as role models, educators are provided with a script that necessitates teaching at the bedside and encourages the setting of expectations and teaching priorities for both preceptors and learners.^20^

#### M - Meeting

First, preceptors are recommended to schedule a meeting with learners to understand responsibilities and educational objectives.

#### i - Behaviors for teachers

The “i” section of the MiPLAN details teacher behaviors at the bedside for efficient and respectful interactions.

- Introductions: the team and purpose are explained to the patient
- In the Moment: preceptors stay focused during the learner’s oral presentation
- Inspection: preceptors demonstrate patient observation through visual physical examination.
- Interruptions: these are minimized during the presentation
- Independent Thought: preceptors encourage an understanding of clinical reasoning

#### PLAN - Patient care, Learner’s questions, Attending’s agenda, Next steps

The PLAN portion of the teaching model establishes teaching priorities for the teacher that allows the highest-yield teaching priorities to take precedent. Educators are not expected to teach to all four priorities but to select a single element as the focus.

### Literature Review

The MiPLAN teaching model has been cited in a single descriptive study.^20^ There have been no further publications to date detailing its generalizability.

### Recommendation

MiPLAN emphasizes the importance of taking the teaching encounter to the bedside. Educators role model professional behaviors in front of learners and include patients in discussions of their own health care plans. Teaching priorities progress from patient care issues, to learners’ uncertainties, and the educator’s agenda. Finally, the MiPLAN teaching model emphasizes the need for setting expectations, an essential habit of skilled educators, to facilitate high-yield education in clinical settings.

## ED STAT!

### Description

Emergency Department Strategies for Teaching Any Time (ED STAT!) was developed in response to a lack of a teaching tool designed specifically for the unique ED environment. This contrasts with OMP and SNAPPS, which were originally created for an ambulatory care setting, and MiPLAN which was originally created for an inpatient care setting. The first 2 steps are best performed at the initial preceptor-learner meeting. The remaining 4 steps are best completed during an individual teaching encounter.^21^

#### Expectations

This is an orientation to the ED. Preceptors should be clear about how they and the learners will work together. This step is especially important for students. For example, preceptors may feel comfortable with learners “cherry-picking” cases or may prefer a certain oral presentation format. By clarifying expectations on both sides, preceptors create enhanced teaching conditions.

#### Diagnose the Learner

Knowing the learner’s objective makes it easier to provide relevant teaching. Asking “What types of cases do you find challenging?” and “What would you like me to provide feedback on today?” provides insights into the learner’s cognitive and behavioral levels.

#### Set-Up

Using the specific patient care scenario, the preceptor poses a question that will be used as the basis for a teaching point. To focus on medical decision-making, a preceptor could ask “if this patient has right upper quadrant pain, what are 5 important diagnoses?” If the focus is on resource utilization, a pertinent question may be “is this a fracture you would splint yourself rather than consulting orthopedics?”

#### Teach

Incorporate strategies for effective teaching. Teaching points should be high-yield, concise, relevant to the learner, generalizable to similar patient cases, and evidence or experience based. Using a repository of resources such as Free Open Access Medical Education can be a powerful supplement. If a patient has an interesting physical exam finding, with the patient’s permission, summon all learners to the bedside for a demonstration, and encourage active learning.

#### Assess and Give Feedback

Constructive nonjudgmental feedback, based on direct observation of the learner, is most valuable. It is important to involve the learner in the feedback process by including a self-assessment, where the learner asks the questions of “what did I do well today” and “what could I improve upon for next time?” This self-assessment can also be used as a foundation for preceptor feedback.

#### Teacher Always (Role Model)

The learner is always watching and learns a great deal implicitly. Preceptors should be aware of body language, verbal, and nonverbal communication always. When teaching, preceptors should acknowledge if statements reflect facts or opinion.

### Literature Review

To date, there is one study that evaluated the impact of ED STAT!, finding that preceptors self-reported an increased amount and quality of teaching as well as an increased confidence level with teaching.^22^

### Recommendation

ED STAT!’s greatest strength is that it is tailored specifically for the ED teaching environment. There are no other specific ED teaching models reported in the literature. The mnemonic is easy to remember and can be used with a variety of clinical cases. It is not necessary to use the model with every single case. Since this is a recently described teaching model, it may require more faculty development.

## AUNT MINNIE

### Description

Aunt Minnie is the technique of pattern recognition. Across the room, just by her hat, dress, or manner of walking, your “Aunt Minnie” is immediately identifiable, even without viewing her face. This technique is rapid and well suited for classic textbook clinical presentations. Described for use in an outpatient setting,^23^ learners provide the chief complaint and a presumptive diagnosis as the entire presentation. The preceptor then sees the patient, corroborating or correcting the learner’s impression. It facilitates exposure of learners to multiple patients in a busy clinical environment as learners rapidly identify representative clinical presentations.

With decision making, System One is heuristic and System Two is deliberate.^24^ Aunt Minnie is System One thinking made explicit, with potential for cognitive error. However, experienced clinicians frequently rely on effective heuristics. Pattern recognition is a learned skill, ideally based on exposure to multiple reinforcing examples. Improved diagnostic accuracy has been demonstrated from reinforcing the value of both analytic and nonanalytic reasoning strategies to learners.^25^

### Literature Review

Aunt Minnie has not been studied.

### Recommendation

Aunt Minnie helps teach pattern recognition. Its benefits include applicability to the busy and diverse ED clinical environment and the opportunity for learners to build a mental database of representative disease presentations. This type of rapid identification can be easily balanced with other more deliberate models.

## SPIT

### Description

In contrast to the pattern recognition of Aunt Minnie, SPIT is a diagnostic tool that encourages learners to broaden their differentials. The mnemonic stands for:

S = Serious
P = Probable
I = Interesting
T = Treatable

A learner may SPIT any time during a patient’s presentation, identifying a serious, probable, interesting, and treatable diagnosis based on initial chief complaint, upon review of nursing records, after evaluating the patient, or when diagnostic results are available. It is often rewarding to see the list evolve as additional information is gathered. SPIT is an engaging and quick technique. Additional discussion is generated by comparing the learner’s and preceptor’s choices. While original authorship for the technique is unclear, it has been promoted by Dr. Judy Paukert.

### Literature Review

SPIT has not been studied.

### Recommendation

SPIT is best utilized as an instructional method for organizing and expanding a learner’s differential diagnosis by a stratified means. It is repeatable as new information arises. SPIT is especially useful in the ED where the learner is expected to focus on “worst first” and consider life threatening diagnoses, while remaining aware of unusual clinical possibilities.

## ACTIVATED DEMONSTRATION

### Description

This technique is most useful for a skill or procedure unfamiliar to the learner. It closely resembles the education adage of “see one, do one, teach one.” The novice learner is asked to watch out for specific aspects or steps while observing the procedure. The more experienced learner should verbalize the steps of the procedure and take a more active role. The senior learner may supervise others and focus on more advanced techniques or potential complications. After the demonstration, the preceptor “activates” the learner by debriefing, for example by asking the learner to describe what was observed or how they would adjust their technique in the future.

### Literature Review

A few papers have described the technique in general^1^ and for adaptation to specific groups such as in outpatient pediatrics,^26^ critical care,^27^ medicine,^28^ teaching healthcare disparities,^29^ family medicine,^30^,^31^ emergency medicine^32^,^33^ and for faculty development.^34^,^35^ Two studies have demonstrated a measurable impact of activated demonstration. An ambulatory teaching workshop trained 128 participants to use this strategy, finding improvements in learner-focused teaching on a post-test.^35^ Another study found statistically significant improvements during a post-intervention Observed Structured Teaching Exercise workshop for teaching senior residents leadership and physical exam skills, using activated demonstration as one of the objectives.^34^

### Recommendation

Activated demonstration is a well-established teaching technique that uses explicitly identified goals and teaches to the learner level. It is an easy way to brief and debrief with the learner. It is best used for teaching a skill or a procedure compared to other models which more broadly cover a clinical encounter.

## CONCLUSION

Seven clinical teaching models were presented which target different types of clinical encounters, and require variable amounts of facilitator and learner preparation. OMP is the best studied of the models and focuses on learner commitment and feedback. SNAPPS is both learner-centric and learner-driven, stressing uncertainty and questioning by the learner. MiPLAN emphases teaching at the bedside in front of the patient. ED STAT! focuses on identifying the learner’s gap and role modeling. Aunt Minnie is a pattern recognition technique ideal for busy shifts with classic presentations. SPIT offers the opportunity to expand differentials multiple times during a teaching encounter. Activated Demonstration involves the learner with specific goals during participation of a skill. An element of faculty development is always important to the success of introducing and practicing teaching strategies, but this review can serve as a template to help educators become more adept and flexible when teaching within the ED. With this armamentarium of clinical teaching tools, today’s educator is well prepared to take advantage of the teachable moment.

## Supplementary Information



## Figures and Tables

**Table:** Summary comparison of learner-centered clinical teaching models.

Model	Overview of technique	Core educational theory	Strengths	Limitations
One-Minute Clinical Preceptor/Microskills	Get a commitment Probe for supporting evidence Teach general rules Reinforce what was done right Correct mistakes Identify next learning steps	Cognitivist Behaviorist Constructivist	Best studied Learner centered/Preceptor driven Easy to learn Teaches higher level concepts Links clinical teaching and patient care Promotes feedback	Not suited for resuscitations or critical time situations
SNAPPS	Summarize Narrow Analyze Probe Plan Select	Cognitivist Humanist	Learner centered/Learner driven Greater interaction between preceptor and learner	Training required for both preceptor and learner
MiPLAN	Meeting i: introductions, in the moment, inspection, interruptions, independent thought Patient care Learner’s questions Attending’s agenda Next steps	Behaviorist Constructivist	Emphasizes role modeling Fosters bedside teaching Highlights importance of setting expectations	Developed for inpatient ward settings not the ED
ED STAT!	Expectations Diagnose the learner Set-up Teach Assess and give feedback Teacher always (role model)	Behaviorist Constructivist	Designed specifically for ED clinical environment Highlights importance of setting expectations	Training required for the preceptors
Aunt Minnie	Pattern recognition	Cognitivist	Well suited for ED clinical environment	Not studied Potential for overemphasis on System I thinking and premature diagnostic closure
SPIT	Serious Probable Interesting Treatable	Cognitivist	Emphasizes broad differential diagnosis Entertaining for learner	Widely used but not studied
Activated demonstration	Assess student’s relevant knowledge Determine what student should learn from skill demonstration Guidance for student participation during skill demonstration Demonstrate clinical skill Discuss learning points with the student Set the agenda for future learning opportunities	Behaviorist	Best used for a procedure or skill	Not flexible for other aspects of clinical teaching (e.g. differentials)

*ED*, emergency department.
