# Supplementary material for: Taking Advantage of the Teachable Moment: A Review of Learner-Centered Clinical Teaching Models
Source: West J Emerg Med. 2017 Dec 5;19(1):28–34. doi: 10.5811/westjem.2017.8.35277 (PMC5785198; doi:10.5811/westjem.2017.8.35277)
Supplement: Supplementary file 1 [file wjem-19-28-s001.docx]

**Appendix.** Case-based Examples Comparing Teaching Models

**OMP**

Learner: This is a 54-year old man with severe right knee pain, abrupt in onset.  It woke him from sleep last night but he was well yesterday before he went to bed.  There is no history of trauma.  His past medical history is remarkable for hypertension for which he takes Lisinopril, diabetes for which he takes Metformin and he has had surgery for appendicitis.  Vital signs are normal.  His knee is red and warm and I think that I can feel an effusion.  It’s diffusely tender and he has very limited range of active motion.  Even when I test passive range of motion, it causes him a lot of pain.

**Get a Commitment**

Preceptor: Do you think that his knee is infected?

Learner: I’m not sure if this is an infection, but I doubt it.  I don’t see any portal of entry to suggest an infection, and he was apparently well before this episode started.  I’m more inclined to think that this is due to gout or pseudogout, but I thought that gout occurred in the first MTP joint.  Given the lack of trauma I doubt fracture, and with the severity of the pain I am less concerned about osteoarthritis.

**Probe for Supporting Evidence**

Preceptor: How are you going to decide if this is gout or not?  Do you need to know for sure?

Learner: I was planning on performing an arthrocentesis to drain the effusion to determine if this is consistent with infection or gout/pseudogout although I have never done a knee tap before.

**Teach General Rules**

Preceptor: No worries, I can walk you through the procedure.  The key to any successful procedure is adequate pain control, proper positioning of the patient, and good aseptic technique.  Having a second person apply pressure to the supra-patellar pouch improves your ability to be successful with the arthrocentesis.  I’ll show you once we have our materials together.

**Reinforce What Was Done Right**

Preceptor: I would agree that your plan to pursue arthrocentesis is the way to go and your concern for the possibility of a septic joint is right on.  I almost always look to tap a red, hot joint unless the presentation is consistent with the patient’s typical prior gout flares.

**Correct Mistakes**

Preceptor: Even without an obvious portal of entry or signs of soft tissue trauma around the knee, I am still concerned about infection.  Septic joints can also be seeded via bacteremia.

**Identify Next Learning Steps**

Preceptor: I heard a recent podcast about the unreliability of uric acid testing in patients with warm, red joints. Let me share it with you.

**SNAPPS**

**Summarize History and Finding**

Learner: This is a 54-year old man with severe right knee pain, abrupt in onset.  It woke him from sleep last night but he was well yesterday before he went to bed.  There is no history of trauma.  His past medical history is remarkable for hypertension for which he takes Lisinopril, diabetes for which he takes Metformin and he has had surgery for appendicitis.  Vital signs are normal.  His knee is red and warm and I think that I can feel an effusion.  It’s diffusely tender and he has very limited range of active motion.  Even when I test passive range of motion, it causes him a lot of pain.

**Narrow the Differential**

Learner: I’m not sure if this is an infection, but I doubt it.  I’m also considering gout and pseudogout.  This is could be simple arthritis or trauma, but there is no mechanism.

**Analyze the Differential**

Learner: It seems that he’s having his third discrete episode of severe pain in this knee.  I don’t see any portal of entry to suggest an infection, and he was apparently well before this episode started.  I think that this is probably gout, but I thought that gout occurred in the first MTP joint.  This is could be an infection but given the acuity of symptoms I think that this is less likely.  Given the lack of trauma I doubt fracture and with the severity of the pain I am less concerned about osteoarthritis.

**Probe Preceptor About Uncertainties**

Learner: Even though I am doubtful of infection, do you think that we should tap the knee?

Preceptor: I almost always look to tap a red, hot joint to evaluate for septic joint unless the presentation is very consistent with the patient’s typical previous gout flares.

**Plan Management**

Learner: I’ll plan on performing an arthrocentesis and drain the effusion to determine if this is consistent with infection or gout/pseudogout.  We’ll likely end up initiating treatment once the results of the tap are back, but I’ll give him some pain medications in the meantime.

Preceptor:  I would agree that your plan to pursue arthrocentesis is the way to go and your concern for the possibility of a septic joint is right on.

**Select Case-Related Issues for Self-Study**

Learner: I’m interested in the utility of uric acid testing in the ED and if it is useful for predicting the presence of gout in acute presentations or in determining outcomes of treatment.  I’ll look this up and get back to you at our next shift.

**MiPLAN**

**M: Meeting**

Preceptor: Since we are at the start of our shift together, I would like to quickly touch base to see what we should work on today.

Learner: OK. I have been wanting to come up with a solid work-up and treatment plan from my first interaction with a patient.

Preceptor: That is a reasonable goal for the shift. Why don’t we try to incorporate a quick bedside oral presentation in front of the patient once you have finished your initial assessment. I’ll come into the room to hear the presentation, examine the patient and we can firm up plans at that point. That will be efficient for patient care.

**i: introductions, in the moment, inspection, interruptions, independent thought**

Learner: (Talking to the patient) This is my supervising attending, Dr. ABC, who I would like to update on what we talked about, and your examination. (Proceeding with the presentation) The patient, Mr. Joint, is a 54-year-old man with severe right knee pain. It started last night before he went to bed abruptly. He denies any injuries. His past medical history is remarkable for hypertension on Lisinopril, diabetes on Metformin and appendectomy.  Vital signs are normal.  On exam, I think I can feel an effusion and his knee is tender with redness and warmth.  He has very limited range of active motion.  Even when I test passive range of motion, it causes him a lot of pain.

Preceptor: (The preceptor listens to the oral presentation without interrupting and examines the patient’s knee while the Learner is discussing her physical examination findings)

Learner: In summary, this 54-year-old male most likely has a septic joint. I would like to treat the patient with antibiotics reassess for improvement of his symptoms over the next 24 hours.

**PLAN: Patient care, Learner’s questions, Attending’s agenda, Next Steps**

Preceptor: Any questions in your mind about the case?

Learner: No, I think that it's straightforward.

Preceptor: (Since the Learner does not have a specific question, the attending pursues her agenda which is a patient care issue) Septic joint is a possibility. However, he doesn’t have any fevers and looks well. It could also be other things like gout and that’s higher on my differential. What do you think of doing an arthrocentesis to confirm the diagnosis?

Learner: Seemed like a septic joint to me but you’re right - I need to think about other things in the differential. Should I consult orthopedics?

Preceptor: Let’s work on getting the arthrocentesis done first and then if the results show that it looks like a septic joint then we can involve orthopedics.

Learner: Sounds like a plan. I’ll talk to Mr. XYZ about the plan for the arthrocentesis and get everything set up once we have consent.

Preceptor: Great. Mr. XYZ, do you have any questions about what we are talking about or what the next steps are?

Patient: Not yet but I do want to hear more about what an arthrocentesis is. Thanks for including me in the discussion.

**ED STAT!**

***Phase 1: Setting Expectations and Diagnosing Learners***

**E: Setting Expectations**

Preceptor: What would you like for me to give you feedback on today?

Learner: I guess that I’ve been trying to work on my procedures, especially orthopedic ones.  I’ve been getting evaluations that say that I need to improve my procedural skills. Can we work on that?

Preceptor: Sure, let’s see how the shift goes and we’ll plan on focusing on procedures today.

**D: Diagnosing the Learner**

Preceptor: How do you think that you are doing in terms of performing procedures?

Learner:  Ok, I suppose.  I feel like I have the dexterity and manual skills to perform the procedures and I usually know the steps, but for some reason the procedures don’t go that well.

Preceptor: I see.  Hopefully we’ll get an opportunity to perform a procedure this shift and we can work on this!

***Phase 2: Teaching the Learner***

**S: Setup**

Learner: I have a 54-year-old man with severe right knee pain that is abrupt in onset.  He is diabetic, has no history of trauma, but his knee is red, hot, and has an effusion.  I think that it's gout but it could be infected.  He needs a knee tap.

Preceptor: Great, here’s an opportunity to get some feedback about your procedural skills.  How about you get everything set up and review the steps of the procedures. Then we can do it together.

**T: Teach a focused point**

Preceptor: It looks like you have all the correct equipment and you know the steps based upon our quick review.  The patient doesn’t look to be well positioned for the procedure.  I would make the patient a bit more comfortable in a bed instead of this chair, get a light, and move this patient from the hall into a room for the procedure.  A key to any successful procedure is adequate pain control, proper positioning, and good aseptic technique.

Learner: Sounds good I’ll do those things.

**A: Assess and give feedback**

Learner: That was the smoothest procedure that I’ve done in a long time!

Preceptor: Great. I would agree.  Your improved setup and positioning allowed your knowledge of the steps of the procedure and good technique to shine through.

**T: Teach always as a role model**

Preceptor: Another tip for procedures that helps me be successful is the use of IV pain medications or sedation for particularly painful procedures.  I’ll often give IV fentanyl or midazolam to patients for procedures such as lumbar puncture, or incision and drainage to help them be more comfortable for the procedure.

**Aunt Minnie**

Preceptor: How about saying a brief hello to the patient in Room 24 and telling me what you think?

Learner: The patient has a red, swollen, painful knee. I would consider gout, osteo/rheumatoid arthritis, septic arthritis, or trauma!

**SPIT**

Preceptor: What are the most serious diagnoses that you may be considering in this patient with an acute painful knee?

Learner: Septic arthritis. If this had been a trauma case, maybe knee dislocation with vascular compromise.

Preceptor: What are the most probable diagnoses that you may be considering?

Learner: Gout, osteoarthritis, rheumatoid arthritis, maybe septic arthritis depending on patient’s risks.

Preceptor: What are some interesting diagnoses that you may be considering in this patient with acute knee pain?

Learner: Psoriatic arthritis, pseudogout, hemophilia, Lyme disease

Preceptor: Assuming the worst-case scenario of septic arthritis, how do we treat that condition?

Learner: I would initiate intravenous antibiotics and call the orthopedic surgeon for a washout in the operating room.

**Activated Demonstration**

Preceptor: I saw your 54-year-old patient with the knee effusion. I agree that the patient needs an arthrocentesis because while it is most likely gout, we should evaluate for septic joint since he has never had gout before.  Have you done this procedure before?

Learner: No, I haven’t but I’ve read about it.

Preceptor: Let’s do it together because it’s your first time doing this procedure.  Can you walk me through the steps?

Learner: First we’ll have the patient lie down in a comfortable position and put a towel under the knee. We’ll use sterile technique. After cleaning the skin, I’ll inject a bit of lidocaine. While that kicks in, I’ll attach an 18-gauge needle to a syringe.  Once the skin is numbed, then I’ll use the syringe to enter the knee, pulling back on the plunger until I get joint fluid.  There are a couple of different approaches.  Which way do you prefer?

Preceptor: I personally like the lateral parapatellar approach, and providing adequate pain control helps too. Excellent to hear you describe proper positioning and reinforce the importance of aseptic technique. Let’s get everything set-up.
